# An Account of Some New Instruments for Tying Polypi of the Uterus, Nose, and Ear, and Enlarged Tonsils; with Cases

**Published:** 1839-07

**Authors:** 


Art. I.—*An Account of some new Instruments for Tying Polypi of the Uterus, Nose, and Ear, and enlarged Tonsils; with Cases.* By William Beaumont, Surgeon to the Islington Dispensary.—*London,* 1838. 4to, pp. 35.

This work contains, as its title indicates, an account of the author's inventions for facilitating the removal of polypi by ligature. The instruments first constructed by Mr. Beaumont were intended for tying polypi of the ear alone: but the contrivance, with the requisite modifications, has been since applied to the removal of polypi from other organs. With regard to the employment of the ligature, and the preference now generally yielded to it over the knife, Mr. B. justly remarks, that "in uterine polypi, the circumstances attending the former operation are less repugnant to the patient than those attending the operation of excision," as being less formidable, and not requiring exposure: and, as relates to the nose, where practicable, "it is by far the least painful operation of the two." The application of ligature to polypi of the ear is a new operation; and Mr. B. informs us that he has tied five such, in all of which cases, hearing was more or less perfectly restored. The use of the ligature in enlarged tonsils, Mr. Beaumont thinks, "may have the advantage over excision, of safely allowing the extirpation of the whole tonsil;" but recommends, "if inflammation supervenes, that the tonsil be cut off with curved, probe-pointed scissors, at the part where it is indented by the ligature."

We shall insert an account of the instruments Mr. Beaumont has invented, in his own words.

"The instrument for tying uterine polypi consists, among other parts, of two rami parallel to each other, save that one is slightly curved towards its point, so as to correspond in some measure with the posterior parietes of the vagina, and the more readily to allow the body of a polypus to pass between the rami; which parts of the instrument are temporarily joined together at the handle, the distance between them being capable of increase or diminution according to the size of the polypus to be tied. The curved ramus is solely for the purpose of aiding in the placing of the noose around the pedicle of the polypus, and may be removed from the rest of the instrument and from the vagina, when that is accomplished. The straight ramus, besides assisting in the application of the ligature, is also, with other parts attached to it, the means by which the noose is tightened, and rendered unyielding. This instrument is perhaps rather complex, but it should be borne in mind that it is to accomplish a complex purpose. It is first to carry a noose around the pedicle of a tumour, in a narrow passage; it is then to constrict the pedicle, so far as to strangulate the tumour; and lastly, to jam the running end of the noose in the knot, so as to prevent any elasticity of the pedicle from enlarging the noose." (pp. 9, 10.)

This general description is succeeded by an explanation of the plates (which are very nicely executed) and directions for the use of the instruments; but for these particulars we must refer our readers to the treatise itself. To tie a polypus of the nostril, or meatus externus of the ear, it is requisite that the instrument be on a much smaller scale than that for tying uterine polypi, and a flat probe must be substituted for the finger, in performing the requisite manipulation to place the polypus between the rami of the instrument: and Mr. Beaumont further advises, that "after tying a polypus of the nose or ear, if the noose should not be applied close to the attachment of the growth, that the two ends of the ligature be twisted until the polypus itself should make a turn or two, not so as to be broken off, but that the pedicle, by being twisted, should itself slough off from the part at which it grew." (p. 14.) For tying an enlarged tonsil, one ramus of the instrument is dispensed with, and in its place a three-pronged instrument employed, in the eyes of which the noose is to be confined: but here, again, we must refer to the plate and its accompanying explanation.

The greater bulk of the letter-press is occupied by a detail of cases. The first related is one of large uterine polypus, which appears to have existed, and increased in growth, during two or three pregnancies, causing premature birth of the children, and had even "been felt or seen between the labia for a year and a half before the patient's last confinement!" The constitutional symptoms were in this case urgent. After examination, the body of the tumour was expelled from the vagina, and the pedicle was thus easily brought within reach of the finger. It was found to be about an inch and a half in length, and the size of a man's thumb. The first ligature (of strong platted silk) broke on being tightened, but left a deep groove, on which another noose was successfully applied: the pedicle was then cut through, at its junction to the body of the tumour, with a curved, probe-pointed bistoury. Little or no pain was experienced, but the large jet of blood from the tumour indicated the danger that might have attended the operation of simple excision, performed on the person of a patient already much exhausted and debilitated. A threatening of peritonitis was subdued by mild antiphlogistic measures, and on the sixth day the noose and pedicle were found loose in the vagina: in three weeks the patient was well. Menstruation recurred afterwards at its regular periods. The tumour, which was of fibrous texture, weighed, after maceration, nearly two pounds and a half. A second case, in which the polypus was smaller, was equally successful. The third case was terminated abruptly, by an attack of inflammation of the lungs, which proved fatal. The fourth case was quite successful. Case five is one of nasal polypus: the noose was, in this instance, "passed easily over the tumour, about two inches down the nostril, and there tied:" the ligature parted on the fifth day, the polypus having previously shrunk, and assumed "a brown, slough-like colour." Four cases of polypus of the ear succeed, all of which terminated favorably. The last case related is one of enlarged tonsil, on which the ligature was applied with a slip-knot noose. "The operation occupied but two or three minutes, and for that time gave some pain, which however ceased immediately after, but recommenced in the course of eight or ten hours, and increased by the next morning, when (twenty-four hours after tying the tonsil) I cut it off, with curved, probe-pointed scissors, at the part indented by the noose, so as to remove the ligature as well as the tonsil. The patient was immediately and entirely relieved from pain: he said that he did not feel the incision, and there was no bleeding; the saliva was barely tinged with blood. There followed little or no inflammation, the patient had no recurrence of pain, and in a few days went out of town." Three weeks after the operation, all that remained was a slight hollow in place of the extirpated tonsil.

The success which has crowned Mr. Beaumont's operations is a sufficient testimony of the efficiency of the means he employs; and he therefore justly merits the thanks of the profession for the publicity he has given to his inventions.

